# Royal jelly-derived proteins enhance proliferation and migration of human epidermal keratinocytes in an in vitro scratch wound model

**DOI:** 10.1186/s12906-019-2592-7

**Published:** 2019-07-12

**Authors:** Yan Lin, Qiqi Shao, Meng Zhang, Chenyue Lu, Joy Fleming, Songkun Su

**Affiliations:** 1 College of Bee Science, Fujian Agriculture and Forestry University, Fuzhou, 350002 China; 2 Key Laboratory of RNA Biology, Institute of Biophysics, Chinese Academy of Sciences, Beijing, 100101 China

**Keywords:** Royal jelly, Human epidermal keratinocytes, Major royal jelly proteins, Wound healing

## Abstract

**Background:**

Skin injury is inevitable in daily life. In recent years, with the increasing morbidity of diseases such as diabetes and metabolic disorders, chronic wounds have become a considerable challenge in clinical practice. Royal jelly, reported to have multifarious biological and physiological properties, has been used as a remedy for a variety of wounds since ancient times. However, the active components and mechanisms underlying the wound-healing properties of royal jelly are still largely unknown.

**Methods:**

Water-soluble proteins of royal jelly were fractionated and investigated for the proliferative and migratory effects on human epidermal keratinocytes (HaCaT) in an in vitro wound healing model. The proteins present in bioactive fractions were characterised and quantified using Label-free protein quantification method. The potential functions of these proteins in biological systems were further analysed using bioinformatic tools.

**Results:**

A protein fraction, mainly containing major royal jelly proteins 2 (MRJP2), MRJP3 and MRJP7, stimulated proliferative and migratory activities in HaCaT cells without visible cytotoxicity. It exerted the greatest effects on the growth of HaCaT cells in the first 48 h. Furthermore, when treated with this protein fraction, the closure rates of the in vitro scratch wound were significantly increased. Functional analysis indicated that MRJP2, MRJP3 and MRJP7 were associated with carbohydrate transport and metabolism.

**Conclusions:**

We fractionated the water-soluble proteins of royal jelly and identified one fraction (Fraction 2) that induced both proliferative and migratory effects on a human epidermal keratinocyte cell line. Major royal jelly proteins (MRJP2, MRJP3 and/or MRJP7) were speculated to possess potential wound-healing bioactivity. This is the first report that royal jelly may improve wound closure via MRJP-induced cellular proliferation and migration. These proteins may be valuable lead compounds for the development of novel wound healing medications. Our findings would facilitate better understanding of the wound repair mechanisms of royal jelly.

## Background

Royal jelly, food given exclusively to larvae and queen bees, is synthesised and secreted by the glands located in the hypopharynx and mandible of nurse honeybees. It is composed of 60–70% water, 11–23% carbohydrate, 9–18% protein, 4–8% lipid and 0.8–3% other compounds such as vitamins, salts, amino acids and minerals [1–4]. To date, major royal jelly proteins (MRJPs) consisting of nine members (MRJP1–9) with molecular weight from 49 to 87 kDa have been identified in royal jelly and taken up 83–90% of its total protein content [5].

Many studies have reported that royal jelly possesses a plethora of biological activities, including antimicrobial [6–9], anti-inflammatory [10], anti-tumour [11], antioxidant [12], immunomodulatory [13] and growth-promoting [14] effects. What is more, it has been documented to be used in folk medicine for the facilitation of wound healing since ancientry [15]. Although some studies have indicated that topical application of royal jelly can shorten the period of infected or uninfected wound healing in animal models and even diabetic foot ulcers [16–18], the substances and precise mechanisms associated with wound healing are still poorly investigated. Previous studies merely reported that royal jelly components, especially 10-hydroxy-2-decenoic acid (10-HDA) and defensin-1, might accelerate wound healing through anti-inflammation, promoting synthesis of growth factors, or migration of skin fibroblasts or keratinocytes [15, 19–22]; MRJPs could induce proliferation of several human cell lines [23].

Wound healing generally occurs naturally without any external interference. Nevertheless, if handled improperly or not dealt with promptly, wounds easily become infected. Clinical treatment of wounds may involve cleaning, disinfection, suturing, antibiotic treatment and dermatoplasty. To date, there is no effective medicine or therapeutic method for the treatment of intractable wounds such as gravely infected wounds or diabetic foot ulcers, resulting in significant suffering to patients, seriously affecting their quality of life, and imposing a severe financial burden on both families and society. It is therefore of significant clinical importance to discover pharmacologically-active substances that create conditions conducive to the process of wound healing and to clarify the precise molecular mechanisms related to their actions.

Here, we explored the wound-repairing activity of royal jelly proteins using keratinocytes which are a type of cells prominently spread in epidermis and are critical in wound healing process, particularly in epithelialization. A water-soluble protein fraction mainly consisting of MRJP2, MRJP3 and MRJP7 was found to induce proliferative and migratory effects in human epidermal keratinocytes (HaCaT) without obvious cytotoxicity, implying the potential of MRJPs in the healing of cutaneous wounds. This is the first report that royal jelly may improve wound closure via MRJP-induced cellular proliferation and migration. Our findings facilitate greater understanding of the wound-healing actions of royal jelly and suggest that MRJPs may have potential applications in the treatment of wound healing disorders and diabetic foot ulcers.

## Methods

### Cell culture

A human epidermal keratinocyte cell line (HaCaT cells, DSMZ No. 771) was purchased from DSMZ, Germany, and was routinely cultured in Dulbecco’s Modified Eagle’s Medium (DMEM) (Hyclone, USA) containing 10% foetal bovine serum (FBS) (Hyclone, USA) and 1% penicillin-streptomycin solution (TransGen Biotech, China) at 37 °C in an incubator with humidified atmosphere and 5% CO_2_.

### Royal jelly sample

Fresh royal jelly produced by Fengqiang No. 1 (*Apis mellifera ligustica*) offspring colonies was harvested from a local apiary in Jiangsu Province, P.R. China, and was deposited in a freezer (− 20 °C) prior to use. No permission was necessary for the collection of royal jelly.

### Fractionation of royal jelly

Royal jelly (10 g) was homogenized in 40 ml of phosphate buffered saline (PBS). Extraction of water-soluble proteins: the royal jelly homogenate was vortexed vigorously at intervals then left to stand on ice over a period of 30 min. The supernatant obtained following centrifugation at 10,000×g for 40 min at 4 °C was dialysed against 2 L of PBS for 3 days at 4 °C. The dialysate was further centrifuged as previously, which was followed by lyophilising the final supernatant in a freeze dryer (Thermo Fisher Scientific, USA) and then storing at − 80 °C before use. The concentration of water-soluble proteins was determined using BCA protein assays [24]. Approximately 50 mg of lyophilised protein was reconstituted in 10 ml of deionised water, 500 μl of which was subjected to an ÄKTA™pure system (GE Healthcare, USA) fitted with a Tricorn™ Superdex 75 Increase 10/300 GL high performance column (10 × 300 mm, 9 μm, GE Healthcare, USA). Deionised water (0.7 ml/min) and λ280 nm wavelength were applied for elution and effluent absorbance detection, respectively. Fractions derived from discrete absorbance peaks were collected and lyophilised.

### Electrophoretic analysis of fractions

Fractionated proteins were analysed electrophoretically using 12% SDS-PAGE gels in which Blue Plus Protein Marker (Transgen, China) was included as a molecular weight protein standard. Electrophoresis was followed by staining the gels with Coomassie Brilliant Blue R-250 to preliminarily analyse the protein composition in each fraction.

### MTT cell viability assay

HaCaT cells (100 μl) suspended in DMEM at a density of 5 × 10^4^ cells/ml were seeded onto each well of 96-well plates and incubated at 37 °C for 24 h in a CO_2_ incubator. The medium was replaced with serum-free DMEM for cell starvation for 12 h, and the cells were subsequently treated with royal jelly fractions, bovine serum albumin (BSA, 3.2 μg/ml) or serum-free DMEM (control) for 24, 48 or 72 h. Then, they were incubated for a further 4 h after addition of 10 μl of 5 mg/ml 3-(4,5-Dimethylthiazol-2-yl)-2,5-diphenyltetrazolium bromide (MTT) solution (Beyotime, China). The generated formazan crystals were dissolved in 100 μl of DMSO following removal of the supernatant. Cell viability was determined by the absorbance at 492 nm in an Infinite F50 plate reader (Tecan, Austria), and expressed as % cell viability = absorbance of protein treated cells/absorbance of serum-free medium treated cells × 100%.

### Scratch-wound assays

Cell migration effects were evaluated using scratch-wound assays. Culture-Inserts (ibidi, Germany) were placed in each well of 24-well plates. A volume of 70 μl of HaCaT cells (5 × 10^5^ cells/ml) was seeded in each cell of the Culture-Insert and cells were cultured as monolayers to confluency for about 18 h in a CO_2_ incubator. Culture-Inserts were removed carefully to form an approximately 500-μm-wide cell free gap, and cells were washed twice to remove non-adherent cells. To observe the effects of royal jelly protein fractions on keratinocyte migration, cells were treated with a series of the protein fractions at different concentrations prepared in serum-free DMEM or with serum-free medium (control). The wounded cell monolayer was observed under a phase-contrast microscope (Olympus, Tokyo, Japan) and images (three per well, captured randomly) were taken at 0 and 24 h following the infliction of wounds which was a cell free nick created by Culture-Inserts [25]. Closure of wounds was measured using Image J software (National Institutes of Health, Bethesda, MD, USA). Wound closure rates were expressed as percentages of the wound area closed at 24 h relative to the initial area of the cell-free region at 0 h. Three replications of each treatment were included in each experiment and all experiments were replicated independently three times.

### Identification and quantitative analysis of fractions possessing bioactivity

Label-free protein quantification was performed using an Easy nanoLC 1200 chromatograph (Thermo Scientific, USA) and Q Exactive HF-X mass spectrometer (Thermo Scientific, USA) to identify and quantify the proteins in the fraction displaying bioactivity. Briefly, samples were loaded onto a pre-column (Acclaim™ PepMap™ 100 C18, 2 cm × 100 μm, 5 μm, Dionex, USA) and an analytical column (Reprosil-AQ Pur C18, 15 cm × 150 μm, 1.9-μm particle, Dr. Maisch, Germany) that was eluted with a linear gradient formed from 95% Solvent A (formic acid/water, 0.1/99.9, v/v) + 5% Solvent B (formic acid/acetonitrile (ACN)/water, 0.1/80/19.9, v/v/v) to 10% Solvent A + 90% Solvent B over 60 min. In the Easy-spray ion source, the spray voltage and the heated capillary temperature were set to 3.2 kV and 320 °C, respectively. Proteins were identified by searching the C101SC18010979-uniprot-Apis-mellifera protein database. Percentages of proteins were calculated by the area of absorption peak of each protein relative to that of total protein.

### Functional analysis of the proteins identified in bioactive fractions

The structural features and potential functions of the proteins present in the bioactive fractions were analysed and annotated by InterPro [26], Cluster of Orthologous Groups (COG) [27], Gene Ontology (GO) [28], and Kyoto Encyclopedia of Genes and Genomes (KEGG) [29] databases in conjunction with BLAST search [30].

### Statistical analysis

GraphPad Prism 5.0 software (GraphPad Software Inc., CA, USA) was employed for statistical analysis, in which one-way ANOVA was used for comparisons of variance. Results with *p*-values less than 0.05 were regarded to be statistically significant. Values were expressed as means ± SEM.

## Results

### Isolation and electrophoretic analysis of proteins from royal jelly

**Fig. 1:**
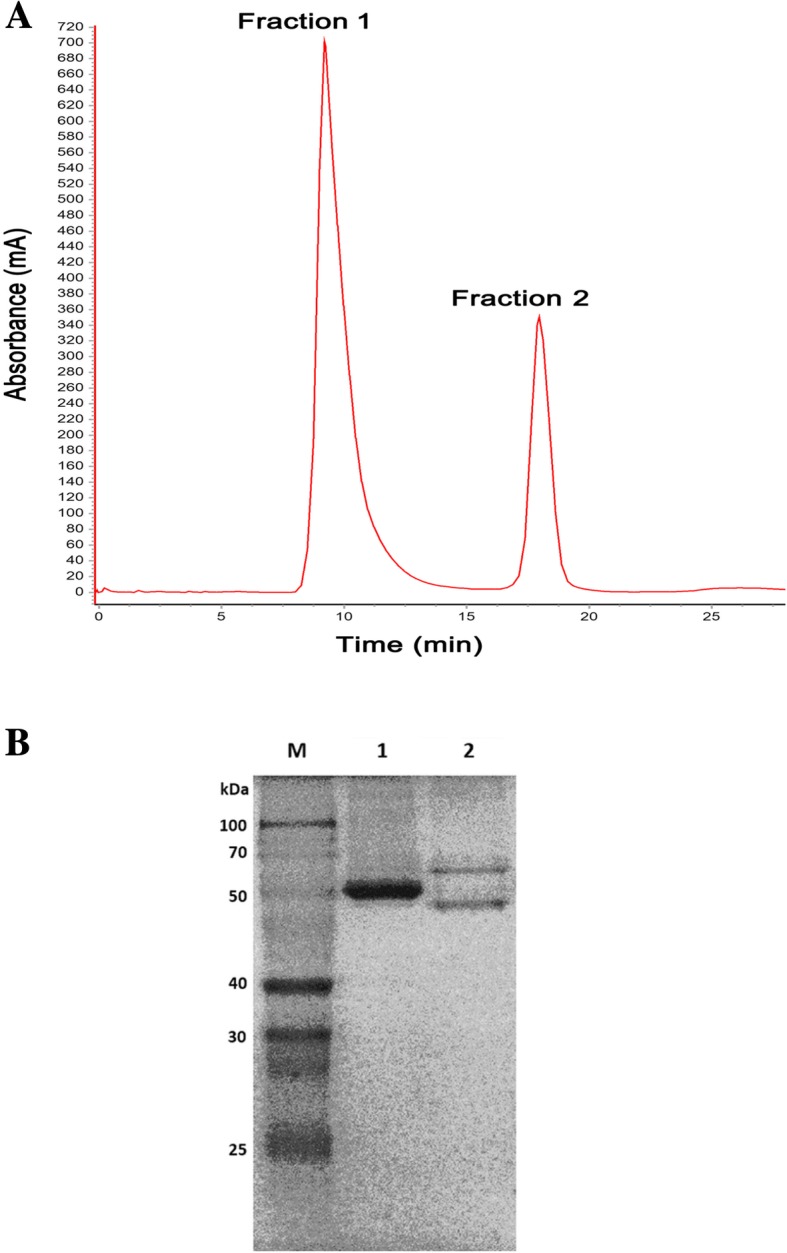
Isolation of proteins from royal jelly. **a** Fractionation of royal jelly water-soluble proteins using an ÄKTA™pure system. The elution positions/retention times of Fractions 1 and 2 are indicated. **b** SDS-PAGE analysis of the protein content of the collected fractions. Lane 1 and lane 2 are the proteins in Fractions 1 and 2, respectively. M represents molecular weight protein standard

Isolation of royal jelly proteins by size exclusion chromatography using an ÄKTA™pure system (GE Healthcare, USA) revealed that two major protein peaks (Fraction 1 and Fraction 2) were eluted at retention times of about 9.5 and 18 min, respectively (Fig. 1a). SDS-PAGE analysis indicated that Fraction 1 was composed of a single protein band with molecular weight of around 55 kDa, and Fraction 2 of two protein bands with molecular weights of 50–60 kDa (Fig. 1b).

### Proliferative effect of protein fractions on human epidermal keratinocytes

**Fig. 2:**
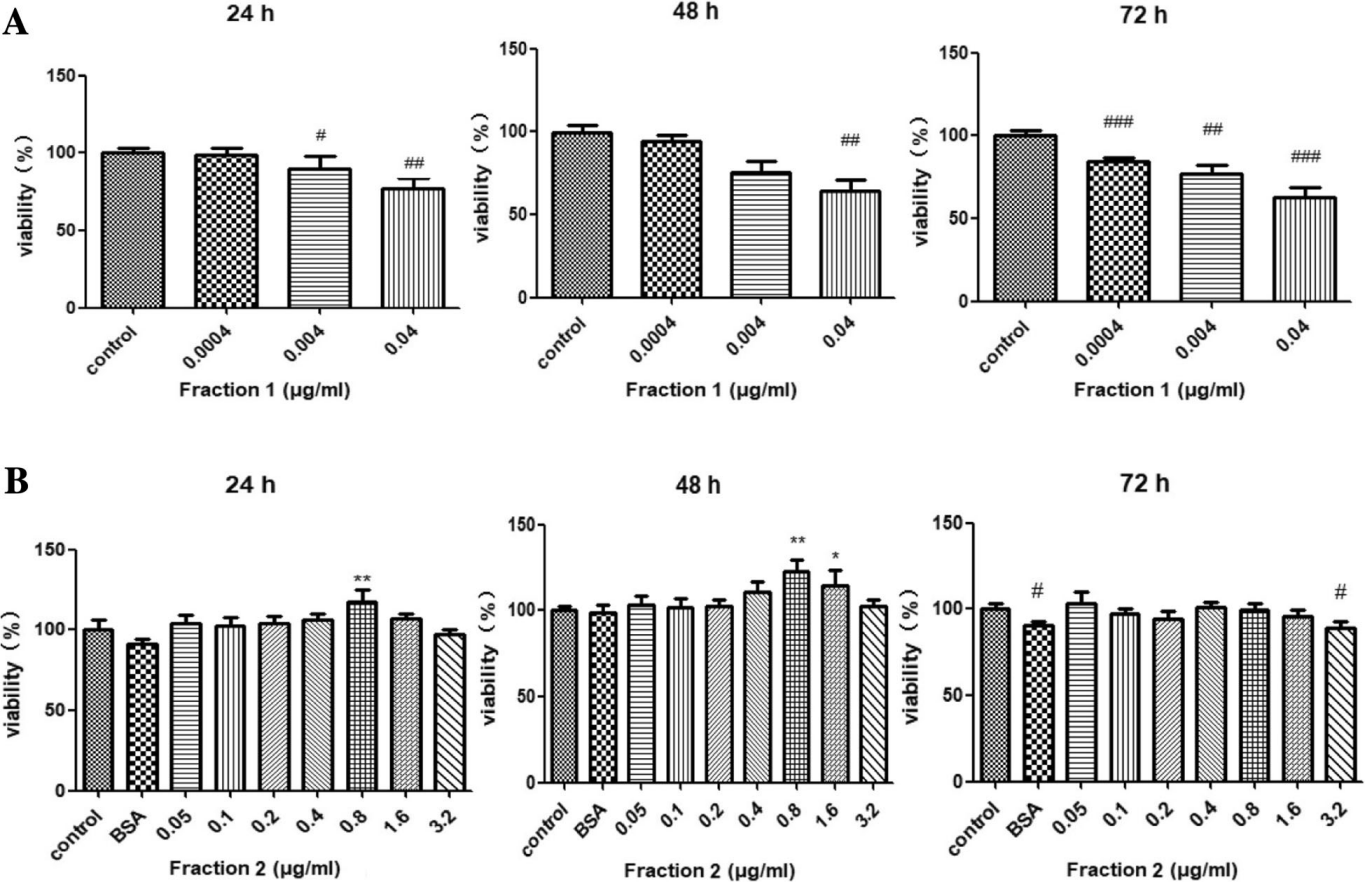
Proliferative effects of protein fractions on human epidermal keratinocytes (HaCaT) after incubation for 24 h, 48 h or 72 h, as evaluated by MTT assays. **a** Proliferative effects of Fraction 1 on HaCaT cells. **b** Proliferative effects of Fraction 2 on HaCaT cells. Results are expressed as the mean ± SEM of at least seven determinations for each test from three independent experiments. Statistical significance of differences: *, *p* < 0.05; **, *p* < 0.01; ***, *p* < 0.001; #, *p* < 0.05; ##, *p* < 0.01; ###, *p* < 0.001 (* and # indicate comparisons with control conditions for proliferative and cytotoxic effects, respectively). BSA, bovine serum albumin, 3.2 μg/ml

The proliferative effect of the royal jelly proteins in these fractions on HaCaT cells was examined. Regardless of the duration of treatment, Fraction 1 was found to be devoid of growth-promoting activity on the cell line at concentrations up to 40 ng/ml and demonstrated severe cytotoxicity at high concentrations (Fig. 2a), which was similar to the effects of MRJP1 (data not shown). By contrast, Fraction 2 exhibited the efficacy of facilitating the proliferation of HaCaT cells at 0.8 and 1.6 μg/ml without causing obvious toxicity at any concentration. When treated with 0.8 μg/ml of Fraction 2, cell growth continued for 48 h (Fig. 2b).

### Cell migratory effects of protein fractions in an in vitro scratch wound model

**Fig. 3:**
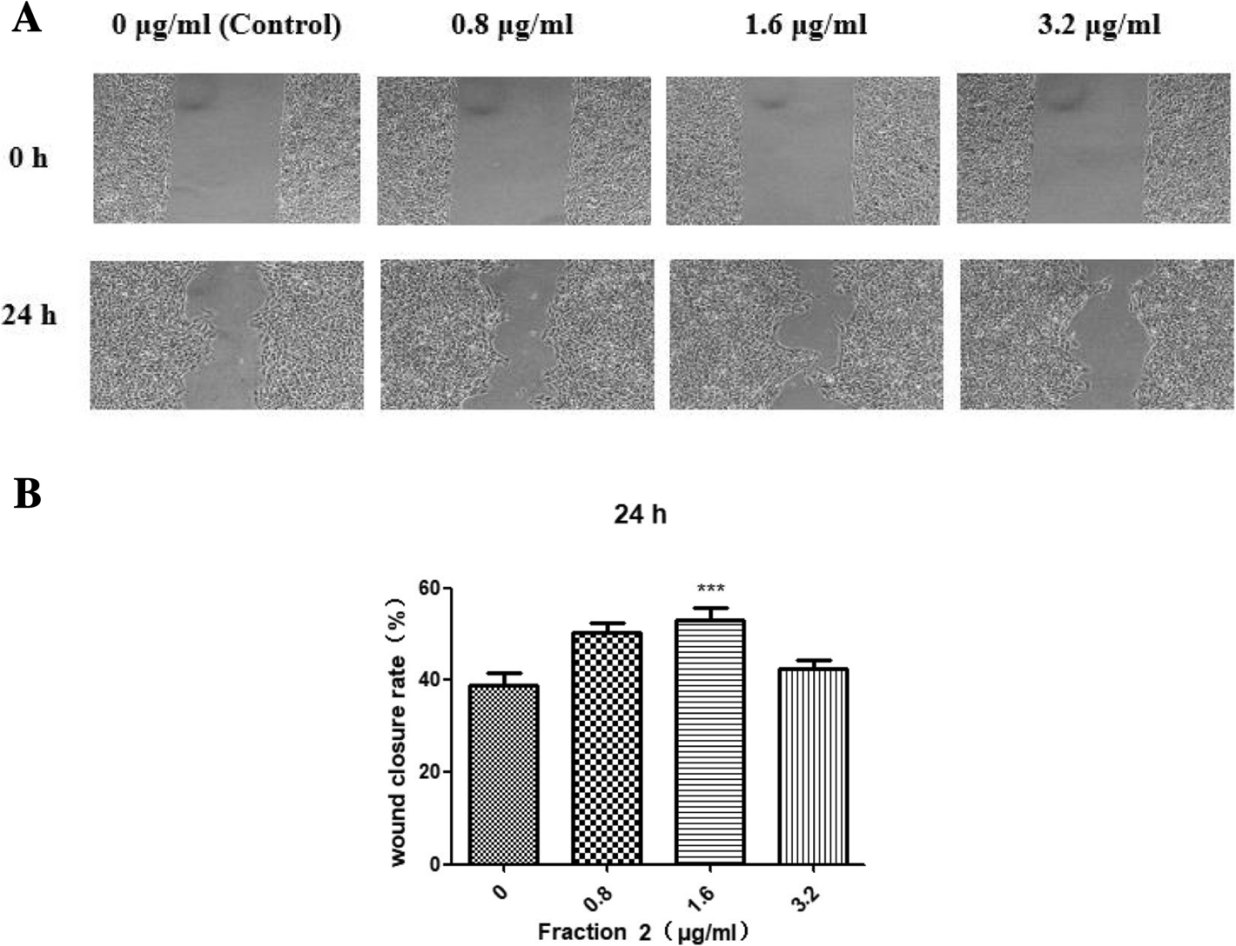
Migratory effects of Fraction 2 on keratinocytes in the in vitro scratch wound assay. **a** Migration of HaCaT cells observed at ×100 magnification, 0 and 24 h post-scratching. **b** Rate of wound healing, as calculated using Image J software. Wound closure rate % = (the scarification area at 0 h – the scarification area at 24 h) / the scarification area at 0 h × 100%. Three randomly captured scarification areas were measured in each well. Results are expressed as the mean ± SEM of at least three determinations for each test from three independent experiments. Statistical significance of differences: *, *p* < 0.05; **, *p* < 0.01; ***, *p* < 0.001 (* comparisons versus control conditions)

Fraction 2, which showed proliferative effects and was nontoxic in keratinocytes, was investigated further for its migratory effects on HaCaT cells in an in vitro scratch wound model. Activity on cell migration was measured as the rate of wound coverage over a period of 24 h. As the micrograph in Fig. 3a, at the end of the 24-h treatment, the scratch wound treated with Fraction 2 at the tested concentrations appeared to be much narrower than the vehicle control. The healing rate of keratinocytes treated with 1.6 μg/ml of Fraction 2 (53%) was dramatically higher than that of the medium-only control at 24 h (Fig. 3b), implying that Fraction 2 could facilitate keratinocyte migration and may possess potential in wound healing.

### Identification and quantification of bioactive protein fractions

**Fig. 4:**
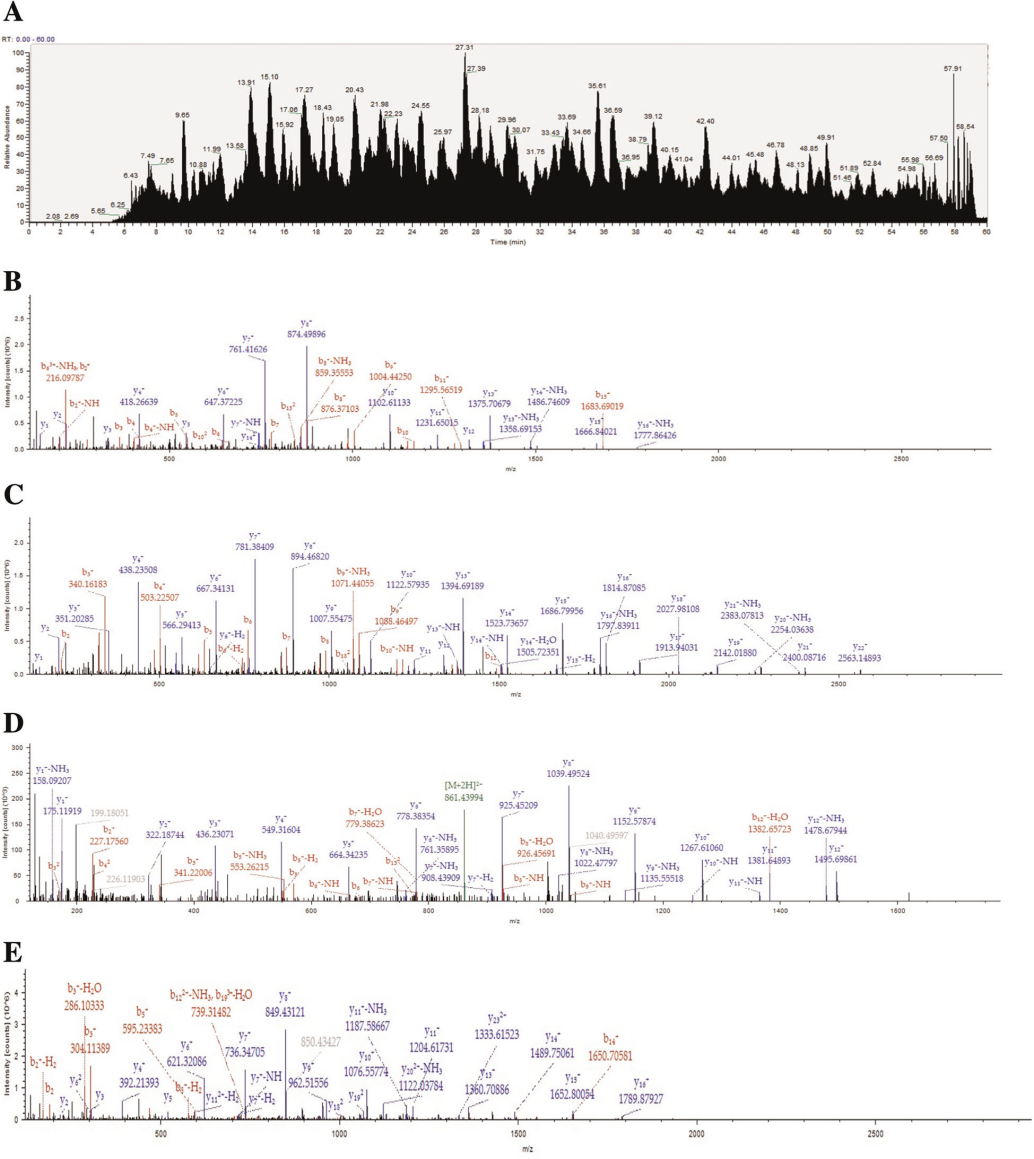
Identification and quantitative analysis of bioactive protein fraction using Label-free protein quantification method. **a** Total ions chromatograph of proteins present in Fraction 2. **b** Partial MS/MS fragmentation sequencing spectrum of MRJP2 (SQFGENNVQYQGSEDILNTQSLAK). **c** Partial MS/MS fragmentation sequencing spectrum of MRJP3 (NPQYEENNVQYEGSQDILNTQSFGK). **d** Partial MS/MS fragmentation sequencing spectrum of MRJP7 (ILNNDLNFNDINFR). **e** Partial MS/MS fragmentation sequencing spectrum of MRJP1 (TSDYQQNDIHYEGVQNILDTQSSAK)

The proteins present in Fraction 2 were successfully identified and quantified using Label-free protein quantification in which absorption peak areas were used for protein quantification and MS/MS fragment ions were used for protein identification. It resulted in identification of a total of 63 proteins including MRJPs, protein kinase and ATP synthase, among which MRJP2 (60%), MRJP3 (30%), MRJP7 (2%), MRJP1 (1%) were the most abundant and characteristic (Table 1). The total ions chromatograph (TIC) of Fraction 2 and the canonical MS/MS fragmentation sequencing spectrums of the identified MRJPs are shown in Fig. 4.

**Table 1:** Identification and quantification of proteins in Fraction 2

Accession	Description	Abundances	Percentages
A0A1Q1N6G0	Major royal jelly protein 2 OS = *Apis mellifera* carnica GN = MRJP2 PE = 4 SV = 1	12095077555	56.443%
D3Y5T0	Major royal jelly protein OS = Apis mellifera GN = MRJP3 PE = 2 SV = 1	6424005583	29.979%
O77061	Major royal jelly protein 2 OS = Apis mellifera GN = MRJP2 PE = 1 SV = 1	788699090.3	3.681%
A0A087ZRA1	Uncharacterized protein OS = Apis mellifera PE = 3 SV = 1	540461377.8	2.522%
Q6IMJ9	Major royal jelly protein 7 OS = Apis mellifera GN = MRJP7 PE = 2 SV = 1	345406314	1.612%
O18330	Major royal jelly protein 1 OS = Apis mellifera GN = MRJP1 PE = 1 SV = 1	202611735	0.946%
A0A087ZW88	Uncharacterized protein OS = Apis mellifera PE = 4 SV = 1	174868784	0.816%
A0A087ZQI5	ATP synthase subunit alpha OS = Apis mellifera GN = Atp5a1 PE = 3 SV = 1	130414480	0.609%
A0A087ZRE3	Elongation factor 1-alpha OS = Apis mellifera GN = EF1a-F2 PE = 3 SV = 1	117294891.1	0.547%
A0A088A436	Tubulin alpha chain OS = Apis mellifera GN = LOC550827 PE = 3 SV = 1	113409161.6	0.529%
A0A088AN20	Uncharacterized protein OS = Apis mellifera GN = RpL40 PE = 4 SV = 1	65715884.16	0.307%
Q4ZJX1	Major royal jelly protein 9 OS = Apis mellifera GN = MRJP9 PE = 2 SV = 1	58901241	0.275%
A0A087ZQ27	Uncharacterized protein OS = Apis mellifera PE = 3 SV = 1	44876322.19	0.209%
A0A088AMB8	ATP synthase subunit beta OS = Apis mellifera GN = Atp5b PE = 3 SV = 1	30241432	0.141%
A0A088AEZ4	Tubulin alpha chain OS = Apis mellifera GN = LOC552766 PE = 3 SV = 1	24948560	0.116%
A0A088A5A6	Uncharacterized protein OS = Apis mellifera GN = LOC409481 PE = 3 SV = 1	20733956.13	0.097%
W8S9B2	Actin (Fragment) OS=Nosema ceranae PE = 3 SV = 1	19727244.75	0.092%
H9KL77	Histone H4 OS = Apis mellifera GN = LOC724757 PE = 3 SV = 1	17945946.88	0.084%
A0A087ZNX0	Uncharacterized protein OS = Apis mellifera GN = Rab11 PE = 4 SV = 1	16626939	0.078%
A0A087ZSC1	Tubulin beta chain OS = Apis mellifera GN = LOC410559 PE = 3 SV = 1	14751532.47	0.069%
A0A088AJJ6	Uncharacterized protein OS = Apis mellifera GN = LOC411989 PE = 3 SV = 1	14168904	0.066%
A0A088AGJ8	Uncharacterized protein OS = Apis mellifera GN = LOC410620 PE = 3 SV = 1	11988349	0.056%
A0A088A5X7	Uncharacterized protein OS = Apis mellifera GN = LOC409167 PE = 4 SV = 1	11909793.5	0.056%
A0A088A3F4	Uncharacterized protein OS = Apis mellifera GN = LOC727045 PE = 4 SV = 1	11490301.06	0.054%
A0A088A2A5	Uncharacterized protein OS = Apis mellifera GN = LOC552272 PE = 3 SV = 1	11100238	0.052%
A0A087ZUL8	Uncharacterized protein OS = Apis mellifera GN = RpS15 PE = 3 SV = 1	10443703	0.049%
A0A087ZR05	Uncharacterized protein OS = Apis mellifera GN = Rpn11 PE = 4 SV = 1	9778002	0.046%
A0A087ZV73	Uncharacterized protein OS = Apis mellifera GN = LOC724873 PE = 4 SV = 1	8411322	0.039%
A0A087ZMS7	Uncharacterized protein OS = Apis mellifera GN = Rab39 PE = 4 SV = 1	7275442.125	0.034%
A0A088A6D6	Uncharacterized protein OS = Apis mellifera GN=Ndufs3 PE = 3 SV = 1	6409224.875	0.030%
A0A088A7D1	Uncharacterized protein OS = Apis mellifera GN = LOC410306 PE = 3 SV = 1	6343857.5	0.030%
A0A088A8F0	Putative H3K9 methyltransferase OS = Apis mellifera GN = 685996 PE = 4 SV = 1	6048775.25	0.028%
A0A087ZYZ1	Tubulin beta chain OS = Apis mellifera GN = LOC408782 PE = 3 SV = 1	6014038.938	0.028%
A0A0B4J2N0	Uncharacterized protein OS = Apis mellifera GN = LOC550794 PE = 3 SV = 1	4973486.5	0.023%
A0A0B4J2L4	Uncharacterized protein OS = Apis mellifera GN = LOC410026 PE = 3 SV = 1	4117948.25	0.019%
A0A088AIY2	Uncharacterized protein OS = Apis mellifera GN = mago PE = 4 SV = 1	4084139.625	0.019%
A0A088AFT2	40S ribosomal protein S6 OS = Apis mellifera GN = LOC725647 PE = 3 SV = 1	3911799.438	0.018%
A0A088ATI7	Uncharacterized protein OS = Apis mellifera GN = LOC409126 PE = 4 SV = 1	3841522.813	0.018%
A0A087ZMT8	Uncharacterized protein OS = Apis mellifera PE = 4 SV = 1	3634688.25	0.017%
A0A087ZZN8	Proteasome subunit alpha type OS = Apis mellifera GN=Prosalpha5 PE = 3 SV = 1	3591316	0.017%
A0A087ZV06	40S ribosomal protein S8 OS = Apis mellifera GN = Rps8 PE = 3 SV = 1	3381020.75	0.016%
A0A088A6T4	Succinate dehydrogenase [ubiquinone] flavoprotein subunit, mitochondrial OS = Apis mellifera GN=SdhA PE = 3 SV = 1	3201888.25	0.015%
A0A088ADQ6	Uncharacterized protein OS = Apis mellifera PE = 3 SV = 1	2594249.063	0.012%
A0A088A2I2	UDP-glucose 6-dehydrogenase OS = Apis mellifera GN = LOC413356 PE = 3 SV = 1	2218352.25	0.010%
A0A088A9V8	Uncharacterized protein OS = Apis mellifera PE = 3 SV = 1	2212138	0.010%
A0A087EPB0	Cell division protein FtsZ OS = Lactobacillus kunkeei GN = ftsZ PE = 3 SV = 1	2057526.938	0.010%
A0A088ANZ0	Uncharacterized protein OS = Apis mellifera GN = LOC551093 PE = 3 SV = 1	1902225	0.009%
A0A088ARA9	60S ribosomal protein L13 OS = Apis mellifera GN = RpL13 PE = 3 SV = 1	1656290.5	0.008%
A0A087EQF2	6-phosphogluconate dehydrogenase, decarboxylating OS = Lactobacillus kunkeei GN = JI66_01835 PE = 3 SV = 1	1644316.875	0.008%
A0A088A2L4	Uncharacterized protein OS = Apis mellifera PE = 3 SV = 1	1338471.375	0.006%
V5 T859	Glyceraldehyde-3-phosphate dehydrogenase OS=Bifidobacterium sp. Bin2N PE = 3 SV = 1	1259643.5	0.006%
A0A088AEV2	Uncharacterized protein OS = Apis mellifera GN = RpL26 PE = 4 SV = 1	1221172	0.006%
A0A087ZW54	Elongation factor Tu OS = Apis mellifera GN = LOC408328 PE = 3 SV = 1	1219225.375	0.006%
A0A088AFM4	Uncharacterized protein OS = Apis mellifera GN = TER94 PE = 3 SV = 1	1214927.25	0.006%
D3JZ08	MRJP5 OS = Apis mellifera PE = 2 SV = 1	802286.1875	0.004%
A0A0B4J2P2	Uncharacterized protein OS = Apis mellifera GN = LOC551386 PE = 3 SV = 1	788959.9375	0.004%
A0A088ATP8	Tubulin alpha chain OS = Apis mellifera PE = 3 SV = 1	654735.875	0.003%
A0A087ZUP0	Uncharacterized protein OS = Apis mellifera GN = 14–3-3epsilon PE = 3 SV = 1	638754.125	0.003%
A0A088AJ01	Mitogen-activated protein kinase OS = Apis mellifera GN = rl PE = 4 SV = 1	616170.75	0.003%
A0A087ZMS5	Uncharacterized protein OS = Apis mellifera PE = 4 SV = 1	579576	0.003%
A0A087EQE2	S-adenosylmethionine synthase OS = Lactobacillus kunkeei GN = metK PE = 3 SV = 1	554618.25	0.003%
A0A088A9W4	Uncharacterized protein OS = Apis mellifera GN=Flo1 PE = 3 SV = 1	333982.2188	0.002%
A0A088ANC5	APD-3-like protein; Apidermin 1-like protein; Apidermin 3-like protein OS = Apis mellifera GN = apd-3 PE = 4 SV = 1	332523.6875	0.002%

### Bioinformatic analysis of the proteins identified in fraction 2

**Fig. 5:**
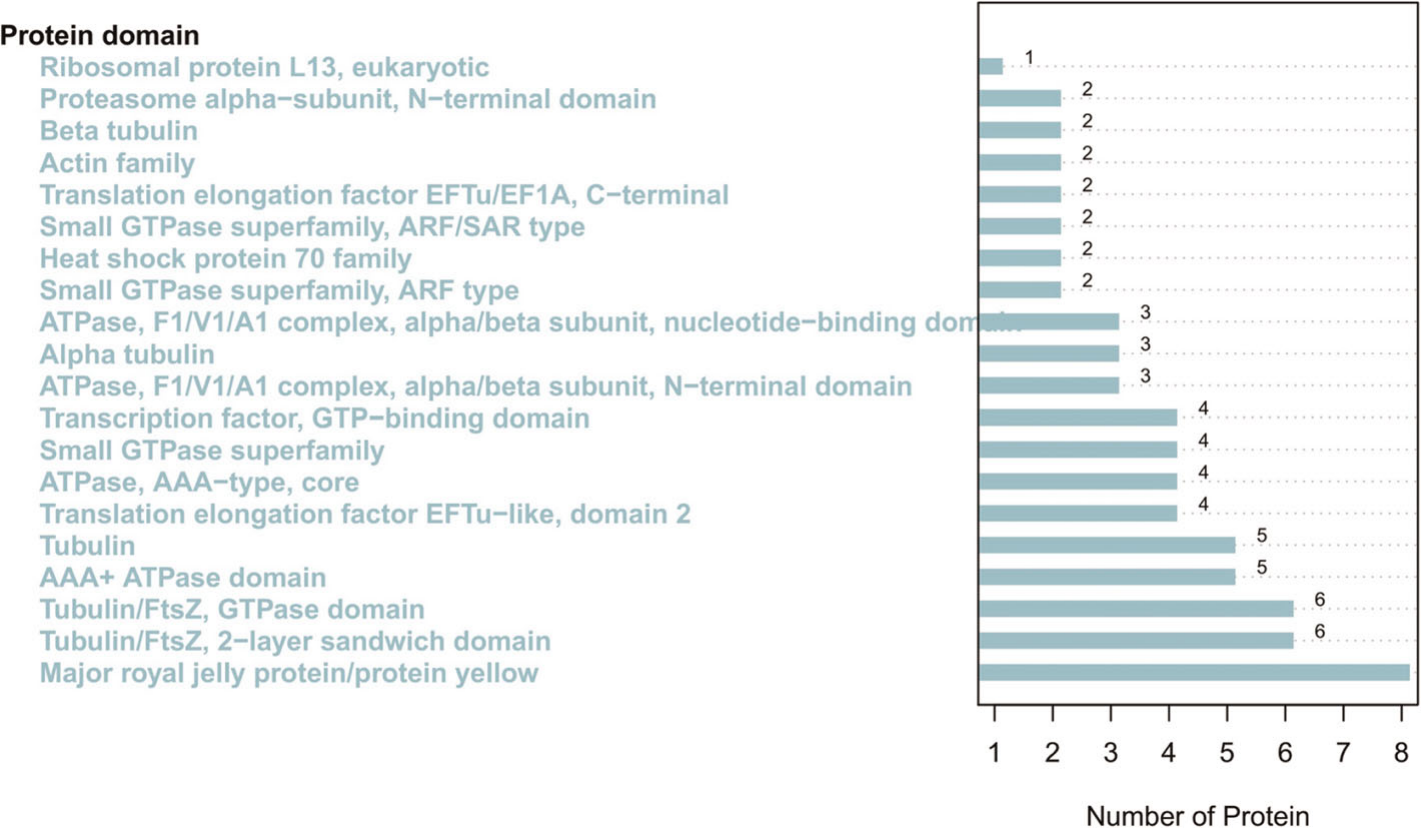
InterPro protein domain classification of proteins identified in Fraction 2

**Fig. 6:**
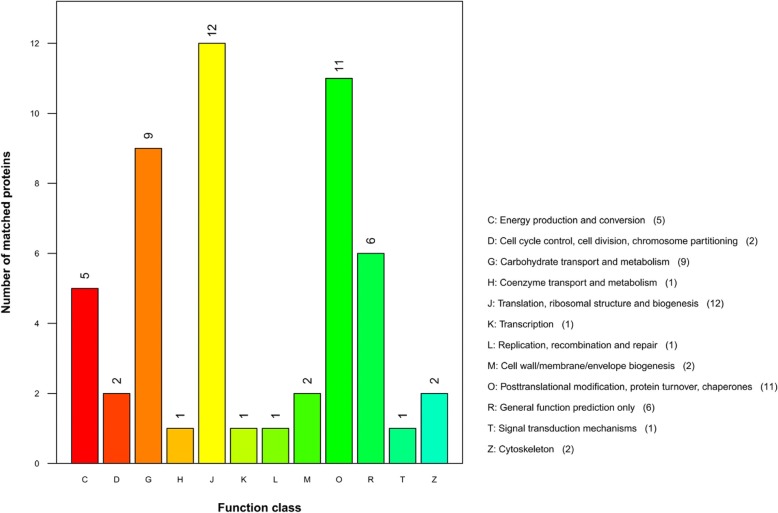
COG function classification of proteins identified in Fraction 2

**Fig. 7:**
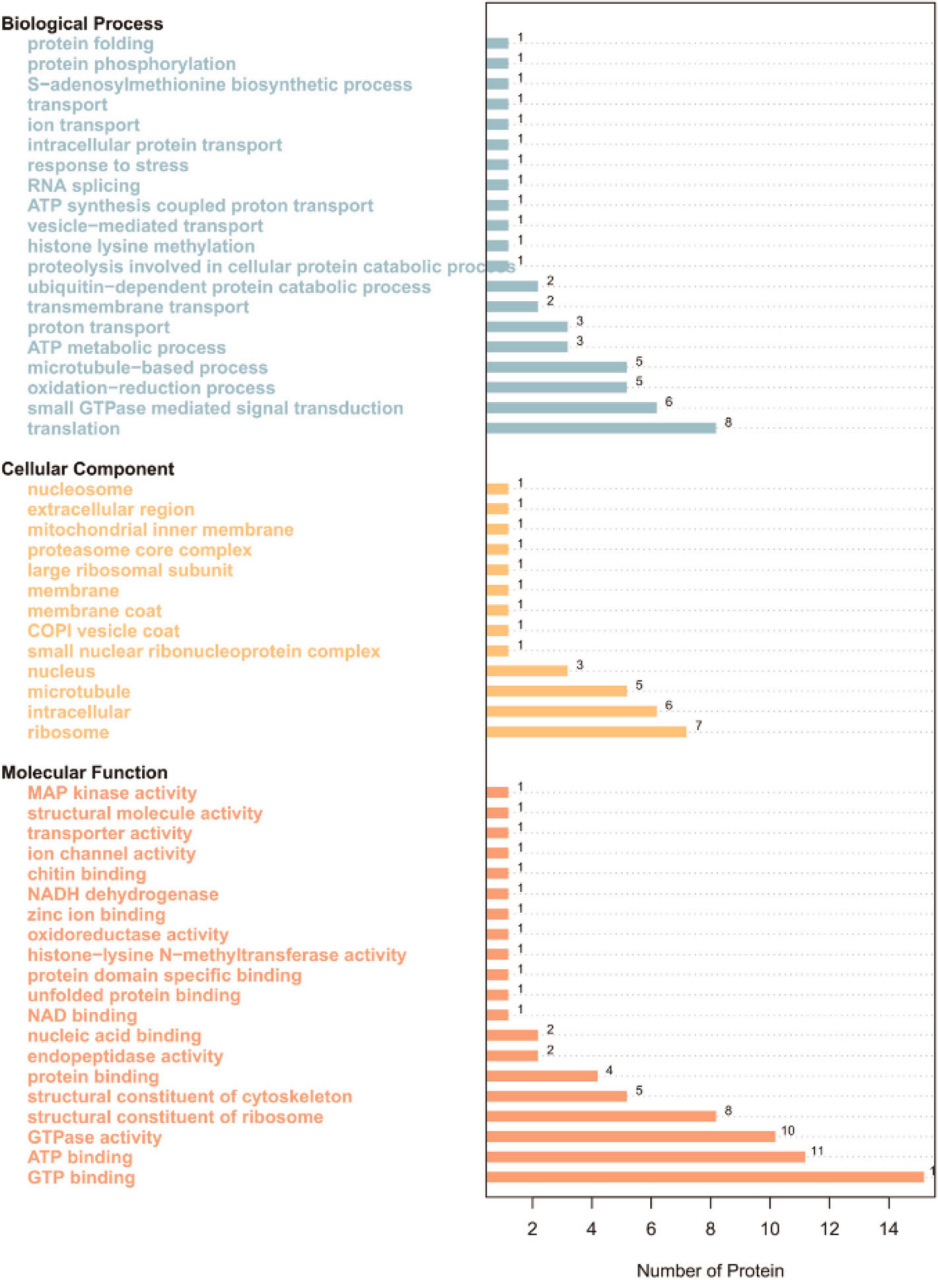
GO functional annotation of proteins identified in Fraction 2

**Fig. 8:**
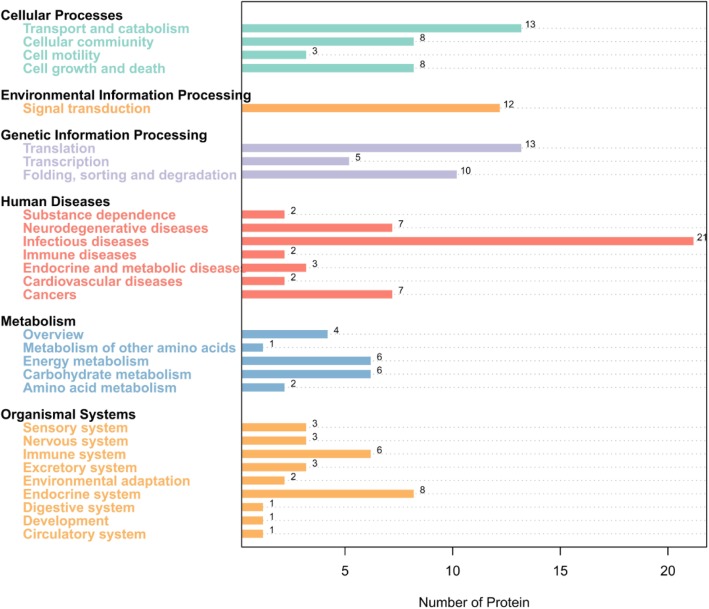
KEGG pathway analysis of proteins identified in Fraction 2

InterPro protein domain classification revealed that the majority of proteins in Fraction 2 shared the typical domain with major royal jelly proteins (Fig. 5), which was consistent with the mass spectrometric results. According to the COG functional annotation, most proteins were related to translation, ribosomal structure and biogenesis (function class J), posttranslational modification, protein turnover, chaperones (function class O), and carbohydrate transport and metabolism (function class G) (Fig. 6 and Table 2), suggesting that these proteins might promote wound healing through the enhancement of protein synthesis. The GO analysis interpreted the roles of proteins in cells, indicating that the most enriched proteins contributed to the translation-associated biological process (Fig. 7). The KEGG pathway analysis illustrated the potential functions and metabolic procedures of proteins in biological system. In accordance with COG and GO analysis, many proteins were concerned with transport and catabolism (cellular processes), and translation (genetic information processing) (Fig. 8). It is noteworthy that masses of proteins (21 proteins) were in connection with human diseases such as Non-alcoholic fatty liver disease, Alzheimer’s disease, Parkinson’s disease and HTLV-I infection.

**Table 2:** COG functional classification of proteins identified in Fraction 2

Protein ID	Identity	E value	COG gene ID	COG num	Functional description	Functional class	Class description
A0A088A2A5	0.62	8.00E-51	YP_875383	COG0048	Ribosomal protein S12	J	Translation, ribosomal structure and biogenesis;
A0A088A2I2	0.64	0	YP_003548756	COG1004	UDP-glucose 6-dehydrogenase	M	Cell wall/membrane/envelope biogenesis;
A0A088A2L4	0.43	0	YP_634186	COG0326	Molecular chaperone, HSP90 family	O	Posttranslational modification, protein turnover, chaperones;
A0A088A3F4	0.32	1.00E-05	YP_002346601	COG1530	Ribonuclease G or E	J	Translation, ribosomal structure and biogenesis;
A0A088A461	0.3	3.00E-17	YP_001958455	COG1196	Chromosome segregation ATPase	D	Cell cycle control, cell division, chromosome partitioning;
A0A088A5A6	0.41	6.00E-37	YP_001737337	COG1100	GTPase SAR1 family domain	R	General function prediction only;
A0A088A5X7	0.34	1.00E-159	YP_001736560	COG0480	Translation elongation factor EF-G, a GTPase	J	Translation, ribosomal structure and biogenesis;
A0A088A6D6	0.52	8.00E-69	YP_422148	COG0852	NADH:ubiquinone oxidoreductase 27 kD subunit (chain C)	C	Energy production and conversion;
A0A088A6T4	0.64	0	YP_742173	COG1053	Succinate dehydrogenase/fumarate reductase, flavoprotein subunit	C	Energy production and conversion;
A0A088A7D1	0.38	1.00E-34	YP_001737337	COG1100	GTPase SAR1 family domain	R	General function prediction only;
A0A088A8F0	0.49	1.00E-113	YP_004484975	COG5257	Translation initiation factor 2, gamma subunit (eIF-2gamma; GTPase)	J	Translation, ribosomal structure and biogenesis;
A0A088A9V8	0.67	1.00E-178	YP_004616696	COG1089	GDP-D-mannose dehydratase	M	Cell wall/membrane/envelope biogenesis;
A0A088ADQ6	0.6	2.00E-47	YP_686965	COG0100	Ribosomal protein S11	J	Translation, ribosomal structure and biogenesis;
A0A088AEV2	0.46	2.00E-29	NP_579542	COG0198	Ribosomal protein L24	J	Translation, ribosomal structure and biogenesis;
A0A088AFM4	0.48	0	YP_004483987	COG1222	ATP-dependent 26S proteasome regulatory subunit	O	Posttranslational modification, protein turnover, chaperones;
A0A087EPB0	0.72	0	YP_007414867	COG0206	Cell division GTPase FtsZ	D	Cell cycle control, cell division, chromosome partitioning;
A0A087EQE2	0.76	0	NP_814529	COG0192	S-adenosylmethionine synthetase	H	Coenzyme transport and metabolism;
A0A088AFT2	0.37	5.00E-19	YP_001737450	COG2125	Ribosomal protein S6E (S10)	J	Translation, ribosomal structure and biogenesis;
A0A088AGJ8	0.5	0	NP_927210	COG0443	Molecular chaperone DnaK (HSP70)	O	Posttranslational modification, protein turnover, chaperones;
A0A088AJ01	0.3	6.00E-24	YP_008152560	COG0515	Serine/threonine protein kinase	T	Signal transduction mechanisms;
A0A088AJJ6	0.42	1.00E-105	YP_003640197	COG0513	Superfamily II DNA and RNA helicase	L	Replication, recombination and repair;
A0A088AMB8	0.81	0	YP_004357703	COG0055	FoF1-type ATP synthase, beta subunit	C	Energy production and conversion;
A0A088AN20	0.84	6.00E-39	YP_004089966	COG5272	Ubiquitin	O	Posttranslational modification, protein turnover, chaperones;
A0A088ANZ0	0.54	0	NP_276090	COG1155	Archaeal/vacuolar-type H + -ATPase catalytic subunit A/Vma1	C	Energy production and conversion;
A0A088ARA9	0.38	5.00E-07	YP_006863204	COG4352	Ribosomal protein L13E	J	Translation, ribosomal structure and biogenesis;
A0A088ATI7	0.34	8.00E-27	YP_008431664	COG1100	GTPase SAR1 family domain	R	General function prediction only;
A0A0B4J2L4	0.5	1.00E-123	NP_070800	COG1222	ATP-dependent 26S proteasome regulatory subunit	O	Posttranslational modification, protein turnover, chaperones;
A0A0B4J2N0	0.52	1.00E-127	NP_614161	COG1222	ATP-dependent 26S proteasome regulatory subunit	O	Posttranslational modification, protein turnover, chaperones;
A0A0B4J2P2	0.54	1.00E-125	NP_275871	COG1222	ATP-dependent 26S proteasome regulatory subunit	O	Posttranslational modification, protein turnover, chaperones;
**A0A1Q1N6G0**	**0.28**	**1.00E-17**	**YP_004643033**	**COG3386**	**Sugar lactone lactonase YvrE**	**G**	**Carbohydrate transport and metabolism;**
D3JZ08	0.37	2.00E-16	YP_001538809	COG1158	Transcription termination factor Rho	K	Transcription;
**D3Y5T0**	**0.3**	**4.00E-25**	**YP_004643033**	**COG3386**	**Sugar lactone lactonase YvrE**	**G**	**Carbohydrate transport and metabolism;**
A0A087EQF2	0.77	0	YP_007414230	COG0362	6-phosphogluconate dehydrogenase	G	Carbohydrate transport and metabolism;
A0A087ZMS7	0.34	9.00E-19	YP_002463522	COG1100	GTPase SAR1 family domain	R	General function prediction only;
O18330	0.28	9.00E-20	YP_004643033	COG3386	Sugar lactone lactonase YvrE	G	Carbohydrate transport and metabolism;
**O77061**	**0.28**	**1.00E-17**	**YP_004643033**	**COG3386**	**Sugar lactone lactonase YvrE**	**G**	**Carbohydrate transport and metabolism;**
Q4ZJX1	0.3	3.00E-21	YP_004643033	COG3386	Sugar lactone lactonase YvrE	G	Carbohydrate transport and metabolism;
**Q6IMJ9**	**0.3**	**2.00E-20**	**YP_004643033**	**COG3386**	**Sugar lactone lactonase YvrE**	**G**	**Carbohydrate transport and metabolism;**
V5 T859	0.79	0	YP_003986273	COG0057	Glyceraldehyde-3-phosphate dehydrogenase/erythrose-4-phosphate dehydrogenase	G	Carbohydrate transport and metabolism;
W8S9B2	0.37	3.00E-70	YP_003266114	COG5277	Actin-related protein	Z	Cytoskeleton;
A0A087ZMT8	0.63	3.00E-13	YP_008432142	COG0638	20S proteasome, alpha and beta subunits	O	Posttranslational modification, protein turnover, chaperones;
A0A087ZNX0	0.35	1.00E-29	YP_008431664	COG1100	GTPase SAR1 family domain	R	General function prediction only;
A0A087ZQ27	0.51	0	YP_001369341	COG0443	Molecular chaperone DnaK (HSP70)	O	Posttranslational modification, protein turnover, chaperones;
A0A087ZQI5	0.73	0	YP_423504	COG0056	FoF1-type ATP synthase, alpha subunit	C	Energy production and conversion;
A0A087ZR05	0.35	1.00E-17	YP_008797954	COG1310	Proteasome lid subunit RPN8/RPN11, contains Jab1/MPN domain metalloenzyme (JAMM) motif	O	Posttranslational modification, protein turnover, chaperones;
A0A087ZRA1	0.37	1.00E-93	YP_003266114	COG5277	Actin-related protein	Z	Cytoskeleton;
A0A087ZRE3	0.55	0	YP_003669641	COG5256	Translation elongation factor EF-1alpha (GTPase)	J	Translation, ribosomal structure and biogenesis;
A0A087ZUL8	0.51	8.00E-43	YP_003650076	COG0185	Ribosomal protein S19	J	Translation, ribosomal structure and biogenesis;
A0A087ZV06	0.41	8.00E-14	YP_001030947	COG2007	Ribosomal protein S8E	J	Translation, ribosomal structure and biogenesis;
A0A087ZV73	0.33	9.00E-30	YP_008431664	COG1100	GTPase SAR1 family domain	R	General function prediction only;
A0A087ZW54	0.57	1.00E-166	YP_001995997	COG0050	Translation elongation factor EF-Tu, a GTPase	J	Translation, ribosomal structure and biogenesis;
A0A087ZW88	0.35	3.00E-60	YP_007146068	COG3325	Chitinase, GH18 family	G	Carbohydrate transport and metabolism;
A0A087ZZN8	0.42	4.00E-62	YP_003434754	COG0638	20S proteasome, alpha and beta subunits	O	Posttranslational modification, protein turnover, chaperones;

MRJPs and the predicted functions are in bold typeface

## Discussion

Cutaneous wound healing impairment has always been a serious health problem as it may result in ulcers, relapse, amputation and even death. Over the past decades, with the high incidence of diabetes, an increasing number of patients are suffering from one of the most typical complications—foot ulcers. Only about half of all such ulcers can be cured and there is a high risk of relapse. It has been reported that one amputation occurs every 30 s and as many as 2,500 patients undergo some form of amputation every single day [31]. According to the International Diabetes Federation (IDF), the diabetic population worldwide is predicted to increase to 592 million in 2035. At present, suturing and skin-grafting are the primary therapies used to treat deep or large defective wounds, and antibiotic therapies are usually applied to infected wounds. There is still no effective agent that promotes the healing of either intractable wounds or diabetic foot ulcers. The discovery and development of novel wound repairing drugs and treatments is thus of significant importance.

Wound healing is a complicated dynamic process which generally involves three consecutive and interrelated phases — inflammatory reactions, proliferative stage and tissue remodelling [32]. An inflammatory reaction occurs in the early stages of wound formation, in which inflammatory cells phagocytose bacteria and cell debris from necrotic tissue, serving as a form of wound cleaning. Subsequently, fibroblasts proliferate to promote the synthesis of extracellular matrix, while granulation tissue grows at the bottom and edges of the wound, filling up the incisions that will be covered by proliferative and migrating basal cells to form an epithelial monolayer. Keratinocytes then proliferate and move towards the centre of the wound, thereby covering the wound surface and completing wound healing. The proliferation, migration and differentiation of fibroblasts and keratinocytes thus plays a vital role in wound re-epithelialization and reparation [33–36].

In this study, we attempted to evaluate the efficacy of royal jelly proteins on human epidermal keratinocytes to identify bioactive components of royal jelly with wound-healing activity and their mechanisms. Following fractionation of royal jelly water-soluble proteins, SDS-PAGE analysis showed that Fraction 1 was a relatively pure protein with a mass of 55 kDa, most likely MRJP1, and Fraction 2 was a mixture of proteins with molecular weights ranging from 50 to 60 kDa (Fig. 1). Fraction 1 was ineffective in wound healing and was even toxic to the growth of HaCaT cells, while Fraction 2 induced some cell growth (Fig. 2). Compared with being incubated with BSA, cells incubated with Fraction 2 showed a significant increase in cell growth (Fig. 2b), suggesting that the proliferative effects of Fraction 2 were different from the nutritive effects of BSA. In addition, as pure MRJP1 and Fraction 1 had very similar profiles and biological effects on the cell viability of HaCaT (data not shown), Fraction 1 was further confirmed as MRJP1. It was surprising that MRJP1, the most abundant MRJP [37], reported to possess various bioactivities [14, 38, 39] including weak up-regulation of mRNA expression of cytokines (TNF-α, IL-1β and TGF-β) in cultured human primary keratinocytes [40], was found to be devoid of having an effect on HaCaT cell growth. Owing to the toxicity and inefficacy of MRJP1, we only investigated Fraction 2 further for its cell migratory effects on an in vitro scratch wounding model. The proportion of wound healing induced by Fraction 2 (1.6 μg/ml) was significantly higher after 24 h incubation than that of the vehicle control (Fig. 3b). As shown in Fig. 2b, however, 1.6 μg/ml of Fraction 2 had little effect on the growth of HaCaT cells at 24 h; moreover, fraction samples used in the wound scratching assay were reconstituted in serum-free medium which had no effect on cell growth. Therefore, effects of Fraction 2 on cell growth and medium-induced cell proliferation in the wound closure observed can be ignored. Fraction 2 may thus mediate wound healing via keratinocyte migration.

Interestingly, Fraction 2 did not function in a strict dose-dependent manner concerning its proliferative and migration efficacy towards keratinocytes, efficacy declining at higher concentrations (Figs. 2b and 3). This phenomenon might be caused by the antagonistic effect of the complicated compositions within Fraction 2, and thus further purification of the fraction or expression of each component as a pure recombinant protein would be desirable to facilitate further functional investigation. In addition, as the procedure of wound healing is complex, involving various categories of cells, cytokines, growth factors, and many other intracellular/extracellular components [41], it will be important to explore the regulatory effects of these proteins on other types of cells such as macrophages.

Qualitative and quantitative analysis of Fraction 2 revealed that the main biologically-active components were MRJP2, MRJP3 and MRJP7. COG analysis demonstrated that MRJP2, MRJP3 and MRJP7 were associated with carbohydrate transport and metabolism (Fig. 6 and Table 2), which might be beneficial to the conversion into nutrition such as proteins to promote wound healing. However, the precise mechanisms underlying the wound healing activity need to be further investigated to facilitate better understanding of the wound repair functions of royal jelly in the future. This is the first experimental evidence that MRJP2, MRJP3 and/or MRJP7 may possess potential wound healing function, providing valuable lead compounds to be developed into novel wound repairing medications.

## Conclusions

In this study, the potential wound healing activity of royal jelly proteins was first time to be concerned. As a result, MRJP2, MRJP3 and MRJP7 present in a water-soluble protein fraction were speculated to possess potential wound repairing activity. Our finding that MRJPs may modulate wound healing by stimulating keratinocyte growth and migration suggests that these naturally-occurring proteins would be biomaterials with potential for development as novel wound-healing agents.

## Data Availability

The datasets used and/or analysed during the current study available from the corresponding author on reasonable request.

## Abbreviations

10-HDA: 10-hydroxy-2-decenoic acid
ACN: Acetonitrile
BSA: Bovine serum albumin
COG: Cluster of Orthologous Groups
DMEM: Dulbecco’s Modified Eagle’s Medium
FBS: Foetal bovine serum
GO: Gene ontology
HaCaT: Human epidermal keratinocytes
KEGG: Kyoto Encyclopedia of Genes and Genomes
MRJPs: Major royal jelly proteins
MTT: 3-(4, 5-Dimethylthiazol-2-yl)-2,5-diphenyltetrazolium bromide
TGF: Transforming growth factor
TIC: Total ions chromatograph

## References

[1] Melliou E, Chinou I (2005). Chemistry and bioactivity of royal jelly from Greece. J Agric Food Chem.

[2] Sabatini AG (2009). Quality and standardisation of royal jelly. J ApiProduct ApiMedical Sci.

[3] Zhang L, Fang Y, Li R, Feng M, Han B, Zhou T, Li J (2012). Towards posttranslational modification proteome of royal jelly. J Proteome.

[4] Ramadan MF, Al-Ghamdi A (2012). Bioactive compounds and health-promoting properties of royal jelly: a review. J Funct Foods.

[5] Honeybee Genome Sequencing Consortium (2006). Insights into social insects from the genome of the honeybee Apis mellifera. Nature..

[6] Bărnuțiu LI, Marghitas LA, Dezmirean DS, Mihai CM, Bobis O (2011). Chemical composition and antimicrobial activity of royal jelly - review. Sci Pap.

[7] Bílikova K, Huang SC, Lin IP, Šimuth J, Peng CC (2015). Structure and antimicrobial activity relationship of royalisin, an antimicrobial peptide from royal jelly of Apis mellifera. Peptides..

[8] Fratini F, Cilia G, Mancini S, Felicioli A (2016). Royal Jelly: an ancient remedy with remarkable antibacterial properties. Microbiol Res.

[9] Tseng JM, Huang JR, Huang HC, Tzen JTC, Chou WM, Peng CC (2011). Facilitative production of an antimicrobial peptide royalisin and its antibody via an artificial oil-body system. Biotechnol Prog.

[10] Karaca T, Şimşek N, Uslu S, Kalkan Y, Can I, Kara A, Yörük M (2012). The effect of royal jelly on CD3(+), CD5(+), CD45(+) T-cell and CD68(+) cell distribution in the colon of rats with acetic acid-induced colitis. Allergol Immunopathol Madr.

[11] Kimura Y (2008). Antitumor and antimetastatic actions of various natural products. Stud Nat Prod Chem.

[12] El-Nekeety AA, El-Kholy W, Abbas NF, Ebaid A, Amra HA, Abdel-Wahhab MA (2007). Efficacy of royal jelly against the oxidative stress of fumonisin in rats. Toxicon Off J Int Soc Toxinology.

[13] Mihajlovic D, Vucevic D, Chinou I, Colic M (2014). Royal jelly fatty acids modulate proliferation and cytokine production by human peripheral blood mononuclear cells. Eur Food Res Technol.

[14] Kamakura M, Suenobu N, Fukushima M (2001). Fifty-seven-kDa protein in royal jelly enhances proliferation of primary cultured rat hepatocytes and increases albumin production in the absence of serum. Biochem Biophys Res Commun.

[15] Bucekova M, Sojka M, Valachova I (2017). Bee-derived antibacterial peptide, defensin-1, promotes wound re-epithelialisation in vitro and in vivo. Sci Rep.

[16] Abdelatif M, Yakoot M, Etmaan M (2008). Safety and efficacy of a new honey ointment on diabetic foot ulcers: a prospective pilot study. J Wound Care.

[17] El-Gayar MH, Aboshanab KM, Aboulwafa MM, Hassouna NA (2016). Antivirulence and wound healing effects of royal jelly and garlic extract for the control of MRSA skin infections. Wound Med.

[18] Temamogullari FK, Hayat A, Baba F (2007). Comparison of the royal jelly and povidone iodine on wound healing in rabbits. J Anim Vet Adv.

[19] Yang XY, Yang D, Zhang W, Wang JM, Li CY, Ye H, Lei KF, Chen XF, Shen NH, Jin LQ, Wang JG (2010). 10-Hydroxy-2-decenoic acid from royal jelly: a potential medicine for RA. J Ethnopharmacol.

[20] Kim J, Kim Y, Yun H, Park H, Kim SY, Lee KG, Han SM, Cho Y (2010). Royal jelly enhances migration of human dermal fibroblasts and alters the levels of cholesterol and sphinganine in an in vitro wound healing model. Nutr Res Pract.

[21] Park HM, Hwang E, Lee KG, Han S-M, Cho Y, Kim SY (2011). Royal jelly protects against ultraviolet B–induced photoaging in human skin fibroblasts via enhancing collagen production. J Med Food.

[22] Tsuruma Y, Maruyama H, Araki Y (2011). Effect of a glycoprotein (apisin) in royal jelly on proliferation and differentiation in skin fibroblast and osteoblastic cells. Nippon Shokuhin Kagaku Kogaku Kaishi.

[23] Chen D, Xin XX, Qian HC, Yu ZY, Shen LR (2016). Evaluation of the major royal jelly proteins as an alternative to fetal bovine serum in culturing human cell lines. J Zhejiang Univ Sci B.

[24] Smith PK, Krohn RI, Hermanson GT, Mallia AK, Gartner FH, Provenzano MD, Fujimoto EK, Goeke NM, Olson BJ, Klenk DC (1985). Measurement of protein using bicinchoninic acid. Anal Biochem.

[25] Banerjee J, Das Ghatak P, Roy S, Khanna S, Sequin EK, Bellman K, Dickinson BC, Suri P, Subramaniam VV, Chang CJ, Sen CK (2014). Improvement of human keratinocyte migration by a redox active bioelectric dressing. PLoS One.

[26] Mitchell AL, Attwood TK, Babbitt PC, Blum M, Bork P, Bridge A, Brown SD, Chang HY, El-Gebali S, Fraser MI (2019). InterPro in 2019: improving coverage, classification and access to protein sequence annotations. Nucleic Acids Res.

[27] Tatusov RL, Fedorova ND, Jackson JD, Jacobs AR, Kiryutin B, Koonin EV, Krylov DM, Mazumder R, Mekhedov SL, Nikolskaya AN (2003). The COG database: an updated version includes eukaryotes. BMC Bioinformatics.

[28] Ashburner M, Ball CA, Blake JA, Botstein D, Butler H, Cherry JM, Davis AP, Dolinski K, Dwight SS, Eppig JT (2000). Gene ontology: tool for the unification of biology. Nat Genet.

[29] Kanehisa M, Goto S, Sato Y, Furumichi M, Tanabe M (2012). KEGG for integration and interpretation of large-scale molecular data sets. Nucleic Acids Res.

[30] Altschul SF, Madden TL, Schäffer AA, Zhang J, Zhang Z, Miller W, Lipman DJ (1997). Gapped BLAST and PSI-BLAST: a new generation of protein database search programs. Nucleic Acids Res.

[31] Henshaw FR, Twigg SM, McLennan SV (2014). What’s the buzz: bee products and their potential value in diabetic wound healing. J Diabet Foot Complicat.

[32] Martin P (1997). Wound healing-aiming for perfect skin regeneration. Science..

[33] Block ER, Tolino MA, Lozano JS, Lathrop KL, Sullenberger RS, Mazie AR, Klarlund JK (2010). Free edges in epithelial cell sheets stimulate epidermal growth factor receptor signaling. Mol Biol Cell.

[34] Plikus MV, Gay DL, Treffeisen E, Wang A, Supapannachart RJ, Cotsarelis G (2012). Epithelial stem cells and implications for wound repair. Semin Cell Dev Biol.

[35] Sivamani RK, Garcia MS, Isseroff RR (2007). Wound re-epithelialization: modulating keratinocyte migration in wound healing. Front Biosci J Virtual Libr.

[36] Woodley DT, Chen JD, Kim JP, Sarret Y, Iwasaki T, Kim YH, O’Keefe EJ (1993). Re-epithelialization. Human keratinocyte locomotion. Dermatol Clin.

[37] Shen L, Zhang W, Jin F, Zhang L, Chen Z, Liu L, Parnell LD, Lai CQ, Li D (2010). Expression of recombinant AccMRJP1 protein from royal jelly of Chinese honeybee in Pichia pastoris and its proliferation activity in an insect cell line. J Agric Food Chem.

[38] Fan P, Han B, Feng M, Fang Y, Zhang L, Hu H, Hao Y, Qi Y, Zhang X, Li J (2016). Functional and proteomic investigations reveal major royal jelly protein 1 associated with anti-hypertension activity in mouse vascular smooth muscle cells. Sci Rep.

[39] Kamakura M (2011). Royalactin induces queen differentiation in honeybees. Nature..

[40] Majtan J, Kumar P, Majtan T, Walls AF, Klaudiny J (2010). Effect of honey and its major royal jelly protein 1 on cytokine and MMP-9 mRNA transcripts in human keratinocytes: Activation of keratinocytes by honey and its MRJP1. Exp Dermatol.

[41] Breitkreutz D, Mirancea N, Nischt R (2009). Basement membranes in skin: unique matrix structures with diverse functions?. Histochem Cell Biol.

